# Studies on Fungal Cultural Filtrates against Adult *Culex quinquefasciatus* (Diptera: Culicidae) a Vector of Filariasis

**DOI:** 10.1155/2011/147373

**Published:** 2011-10-29

**Authors:** Gavendra Singh, Soam Prakash

**Affiliations:** Department of Zoology, Environmental and Advanced Parasitology and Vector Control Biotechnology Laboratories, Dayalbagh Educational Institute, Dayalbagh, Agra 282 005, India

## Abstract

Entomopathogenic fungi have significant potential to control mosquito population. The culture filtrates of *Fusarium oxysporum, Lagenidium giganteum, Trichophyton ajelloi,* and *Culicinomyces clavisporus* were evaluated against adults of *Cx. quinquefasciatus*. The culture filtrates were obtained by filtering the broth through Whatman-1 filter paper. These culture filtrates of *C. clavisporus* have been found significantly pathogenic with LC_50_-2.5, LC_90_-7.24, and LC_99_-8.7 ML, respectively, after exposure of 24 h. However, the culture filtrates when were combined, in ratios 1 : 1 : 1 of *Fusarium oxysporum, Lagenidium giganteum, Trichophyton ajelloi* the mortalities were significantly increased. The LC_50_-3.71, LC_90_-8.12, and LC_99_-11.48 were significantly recorded after exposure of 10 hrs. Similarly, the culture filtrates of *T. ajelloi, Culicinomyces clavisporus,* and *L. giganteum* have been combined in ratios 1 : 1 : 1. Similarly the LC_50_-1.94, LC_90_-4, and LC_99_-6.16 ML Were recorded after exposure of 10 hrs. The results of present study show promise for the use of selected fungal metabolites for control of *Cx. quinquefasciatus* in the Laboratory.

## 1. Introduction

Fungus entomopathogens show potential as alternative biological control agents against mosquitoes and used as currently developed fast action chemical insecticides [1]. The mosquito pathogenic fungi that target larval instars include the chytridiomycetes *Coelomomyces* [2, 3]. Only few studies have evaluated these pathogens against the adult stage of tropical disease vectors. In adults *Ochlerotatus sierrensis* infected with the deuteromycete *Tolypocladium cylindrosporum*, there was 100% mortality after ten days [4]. Scholte et al. [5] reported that also adults of *An*. *gambiae* were susceptible to *B*. *bassiana*, *Fusarium* spp., and *Metarhizium anisopliae*. 

So far the extracellular secondary metabolites from three hundred and fifty fungi and ninety four actinomycetes have been screened for larvicidal activity against *Cx*. *quinquefasciatus*, *An*. *stephensi,* and *Ae*. *aegypti* [6]. The metabolites of *Chrysosporium tropicum* have been found highly pathogenic as adulticides against *An. stephensi*, *Cx*. *quinquefasciatus,* and *Ae*. *aegypti* [7]. Therefore, the fungi are weapons with great potential in mosquito vector control [8]. Recently, Paula et al. [9] investigated the combinated effect of *M. anisopliae* with the insecticide Imidacloprid increasing the virulence of the fungus against the dengue vector *Ae. aegypti*, whilst the use of entomopathogenic fungi against mosquitoes has provided encouraging results under controlled laboratory conditions [10, 11] and in the field [12]. 

Filariasis is a global public health problem. One hundred and twenty million people are currently infected and around 1.3 billion at risk of infection [13, 14]. However, it has been estimated that the Japanese encephalitis is endemic in one hundred and thirty five districts of India [15]. Hence, the development of fungal metabolites would open a new option to reduce the population of *Cx*. *quinquefasciatus* as vector of these diseases. The purpose of this study was to evaluate the lethal activity of culture filtrates from fungus* F. oxysporum*, *L. giganteum*, *T. ajelloi,* and *C. clavisporus* separately and combined on adults of *Cx*. *quinquefasciatus.*

## 2. Materials and Methods

### 2.1. Fungal Strains

The *Fusarium oxysporum *(MTCC-2485), *Lagenidium giganteum* (MTCC-719), *Trichophyton ajelloi* (MTCC-4878), and *Culicinomyces clavisporus* (MTCC-987) were obtained from Microbial Type Culture Collection and Gene Bank (MTCC), Institute of Microbial Technology, Chandigarh, India.

### 2.2. Preparation of Broth and Cultures

#### 2.2.1. *Fusarium oxysporum* (Schlecht Endahl) and *Trichophyton ajelloi* (Ajelloi)

**Figure 1:**
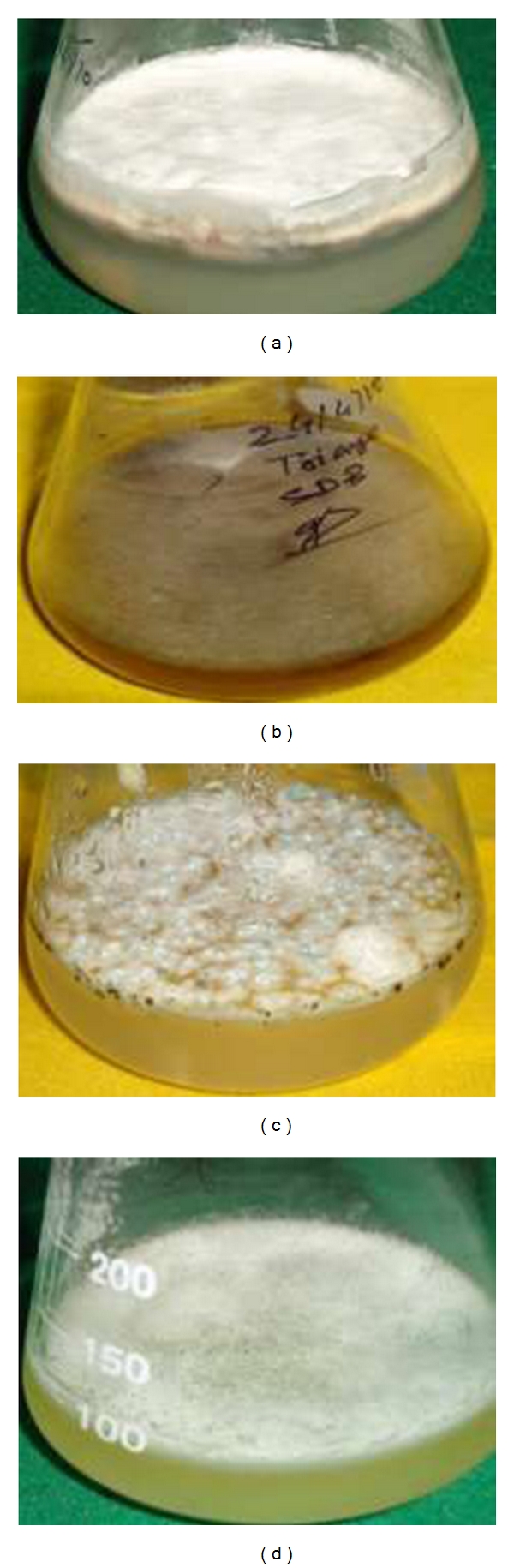
The culture of (a): *Fusarium oxysporum*, (b): *Trichophyton ajelloi*, in Suboraud dextrose broth, (c): *Lagenidium giganteum*, and (d): *Culicinomyces clavisporus* in EmYPss broth maintained in laboratory.

The Subouraud dextrose broth (SDB) was prepared by the method of Gardner and Pillai [16]. Four 250 mL conical flask, each containing 100 mL Suboraud dextrose broth (Dextrose 40 g, peptone 10 g, deionized water 1000 mL) were autoclaved at 20 psi for 20 min. The broth was supplemented with 50 *μ*g/mL chloramphenicol as a bacteriostatic agent.* F*.* oxysporum* colonies grown on the Suboraud dextrose agar plates were transferred to each flask using the inoculation needle. The conical flasks inoculated with *F*.* oxysporum* were incubated at 24 ± 2°C for 15 days (Figures 1(a) and 1(b)). 

#### 2.2.2. *Lagenidium giganteum* (Couch)

Five 250 mL conical flasks each containing 100 mL PYG (Peptone 1.25 g, Yeast Extract 1.25 g, Glucose 3.0 g, and Deionized water 1.0 L) were autoclaved at 20 psi for 20 min. The broth was later supplemented 50 *μ*g/mL chloramphenicol as a bacteriostatic agent. The colonies of *L. giganteum* grown on PYGA plates were transferred to each flask using the inoculation needle. The conical Flasks, inoculated with *L. giganteum*, were incubated at 25°C for 15 days (Figure 1(c)).

#### 2.2.3. *Culicinomyces clavisporus* (Couch, Romney, and B. Rao)

The EmYPss medium was prepared for culture of *C. clavisporus*. Five 250 mL conical flasks each containing 100 mL EmYPss (yeast extract 4 g, soluble starch 15 g, Dipotassium hydrogen phosphate 1 g, Magnesium sulphate 0.5, and Deionized water 1 L) were autoclaved at 20 psi for 20 min. The broth was supplemented 50 *μ*g/mL Chloramphenicol as a bacteriostatic agent. The colonies of *C. clavisporus* grown on EmYPss (yeast extract 2 g, Soluble starch 7.5 g, Dipotassium hydrogen phosphate 0.5 g, Magnesium sulphate 0.5 g, Agar 10 g, Distilled water 500 mL) solid medium plates were transferred to each flask using the inoculation needle. The conical flasks, inoculated with *C. clavisporus,* were incubated at 25°C for 10 days (Figure 1(d)).

### 2.3. Filtration of Extracellular Metabolites and Bioassay

The extracellular metabolites were obtained by filtering the broth through Whatman-1 filter paper. The bioassays were conducted from these metabolites as per the standard methods and protocols of World Health Organization [17]. A total 50 sugar-fed 2–5-day-old female *Cx*. *quinquefasciatus *were used at each concentration for exposure of 24 hrs. The selected concentrations of metabolites were sprayed in cages (25 cm length × 15 cm width × 5 cm depth). Each exposure was done in separate batches in the adults. Similarly the control was run with deionized water to test the natural mortality. Each bioassay including the control was conducted in triplicate on different days.

### 2.4. Statistical Analysis

The relationship between dose and mortality was analysed by probit regression analysis [18]. The LC_50_, LC_90,_ and LC_99_ values were calculated with 95% fiducial limits. If the mortality in the controls was above 5%, results with the treated samples were corrected by using Abbott's formula [19]:

(1) $$\text{Mortality}\;\left(\text{\%}\right)=\frac{X-Y}{100-Y}\times 100,$$

where *X* = the percentage of mortality in the treated sample and *Y* = the percentage mortality in the control.

## 3. Results and Discussion

Fungus and their products are virulent and promising alternative to chemical control of mosquito larvae and adults [12]. The first report of *M*.* anisopliae *IP pathogenicity in adult *An*. *gambiae* and *An*. *arabiensis* has the potential to be a biocontrol agent for African malaria vector species [11]. The results of field study in a rural village in Tanzania revealed that the wild mosquitoes have been infected and reduce life span after resting on 3 m^2^ *M*.* anisopliae* impregnated black cotton sheet suspended from ceilings in traditional houses [12]. 

**Figure 2:**
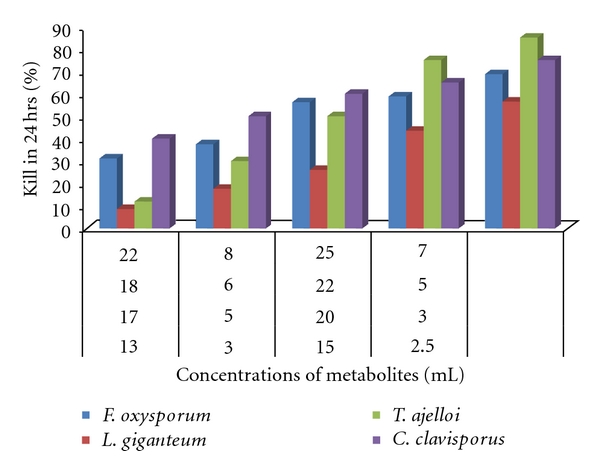
Effect of metabolites of *Fusarium oxysporum*, *Lagenidium giganteum*, *Trichophyton ajelloi,* and *Culicinomyces clavisporus* on *Culex quinquefasciatus. *

However, the present shows that the fungal metabolites have directly sprayed on population *Cx*. *quinquefasciatus*. The metabolites of *F*. *oxysporum* and *T*. *ajelloi* have been found effective with higher concentrations (LC_99_-52.48 and LC_99_-66.06 mL) after exposure of 24 hrs. However, the metabolites of *L*. *giganteum* and *C*. *clavisporus* show significant mortality at lower concentrations (LC_99_-11.3 and LC_99_-8.7 mL) after exposure of 24 hrs (Table 1, Figure 2). A new method, the K bar coating, has been applied as fungal spore suspension onto paper substrates, and coating layers with accurate effective spore concentrations were found effective for adult mosquitoes [20]. The combinations of biopesticides and insecticides treated bed nets could be enhanced for malaria control [21]. Paula et al. [9] for the first time reported that a combination of insecticides and entomopathogenic fungus has been tested against *Ae*. *aegypti*. This study shows the potential of IMI as an alternative to the currently employed pyrethroid adulticide. This study strongly recommended that the *Ae*. *aegypti* could be controlled by surface application of entomopathogenic fungi and that the efficiency of fungi could be increased by combining the fungi with ultralow concentrations of insecticides, resulting in higher mortalities in short exposure of time. 

**Figure 3:**
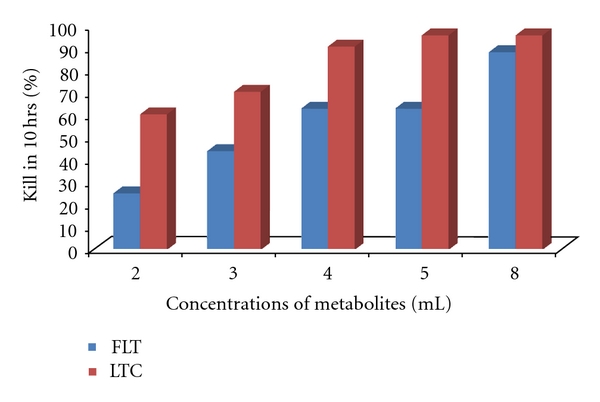
Effect of combine metabolites of *Fusarium oxysporum*, *Lagenidium giganteum*, *Trichophyton ajelloi* (FLT), and *Lagenidium giganteum, Trichophyton ajelloi, Culicinomyces clavisporus *(LTC) on *Culex quinquefasciatus *

In this investigation the effect of combine metabolites should be promoted for control of *Cx*. *quinquefasciatus*. The metabolites of *F*. *oxysporum*, *L*. *giganteum,* and *T*. *ajelloi* were mixed in ratios 1 : 1 : 1; the percent of mortality increases significantly in short time. The LC_99_ of 11.48 was recorded after exposure of 10 hrs. Moreover, the metabolites of *T*. *ajelloi*, *C*. *clavisporus,* and *L*. *giganteum* were applied in ratios 1 : 1 : 1; the percent mortality was highly increased in 10 hrs (Table 1, Figure 3). Our current understanding for adult mosquitoes control effort has focused on existing products and procedures to reduce mortality and morbidity. However, the mosquito control can be achieved with the fungal metabolites. Thus results of present study now accelerate the development of new generation tools and knowledge aimed specifically for filariasis vector. This new strategy of combining different fungal metabolites can be significant approach for controlling certain mosquito species. Moreover, the effects of the combination and insecticides and entomopathogenic fungi have been successfully studied for the control of malaria mosquitoes. Recently, Paula et al. [9] have investigated the possibility of combining an insecticide with an entomopathogenic fungus reducing the vectorial capacity by joint action of the two agents. Mnyone et al. [22] have found that the fungal infection reduced the survival of mosquitoes regardless of their age and their blood-feeding status. The formulations of *M*. *anisopliae* and *B*. *bassiana* can equally affect mosquitoes of different age classes, with them being relatively more susceptible to fungus infection when non-blood-fed. 

The application of *F*. *pallidoroseum* against *Culex* species could reduce the burden of filariasis and Japanese encephalitis in the tropical countries [23]. The fungal efficacy has always been found to be dependent upon the conidial concentration used to infect the mosquitoes. The current methods have a limited ability to control adult mosquitoes. The entomopathogenic fungi are themselves living organisms; it is important to test whether they will survive and be effective under field conditions where the temperature and humidity fluctuate [24]. In laboratory studies the culture filtrates of *L*. *giganteum* and *Culicinomyces clavisporus* have been significantly found pathogenic against *An*. *stephensi*, *Cx*. *quinquefasciatus,* and *Ae*. *aegypti* [25, 26]. The outcome of this study distinctly demonstrates that the combining fugal metabolites induced a higher impact on mosquito survival than the use of these control agents alone. The recorded efficacy shows the potential for integrated fungus control measures to dramatically reduce filarial vector. 

The pathogenic fungi produce a wide variety of toxic metabolites, which vary from low molecular weight products of secondary metabolism to complex cyclic peptides and proteolytic enzymes [27]. A significant progress has been made in understanding enzymes involved with the penetration of host cuticle and the role of mosquitocidal toxins. The combining of fungal metabolites can be more effective by joint action of numerous toxins and enzymes. 

## Figures and Tables

**Table 1:** The lethal concentration (LC) in mL of *T. ajelloi* metabolites on *C. quinquefasciatus* with their confidential limits (CL) values with their probit equations.

Lethal Conc. in mL	*Fusarium oxysporum* 24 hrs	*Lagenidium giganteum * 24 hrs	*Trichophyton ajelloi * 24 hrs	*Culicinomyces clavisporus * 24 hrs	*F. oxysporum *+ *L. giganteum* + *T. ajelloi * 10 hrs	*T. ajelloi *+ *C. clavisporus* + *L. giganteum * 10 hrs
LC_50_-CL	14.79	7.94	20	2.5	3.71	1.94
	(13.62–15.96)	(6.8–9.08)	(18.8–21.2)	(1.46–3.54)	(2.67–4.75)	(0.56–3.32)
LC_90_-CL	29.51	10	36.3	7.24	8.12	4
	(28.34–30.68)	(8.86–11.14)	(35.1–37.5)	(6.12–8.36)	(7.00–9.24)	(2.8–5.20)
LC_99_-CL	52.48	11.3	66.06	8.7	11.48	6.16
	(51.31–53.5)	(10.08–12.36)	(64.75–67.37)	(7.56–9.84)	(10.31–12.65)	(4.82–7.5)
Probit equations	*y* = 0.06 + 4.21*x*	*y* = 0.51 + 6.48*x*	*y* = −0.01 + 4.03*x*	*y* = 0.56 + 8.77*x*	*y* = 0.41 + 8.06*x*	*y* = 0.37 + 10.16*x*
